# Potential of Annatto Seeds (*Bixa orellana* L.) Extract Together with Pectin-Edible Coatings: Application on Mulberry Fruits (*Morus nigra* L.)

**DOI:** 10.3390/polym17050562

**Published:** 2025-02-20

**Authors:** Igor Gabriel Silva Oliveira, Karina Sayuri Ueda Flores, Vinícius Nelson Barboza de Souza, Nathaly Calister Moretto, Maria Helena Verdan, Caroline Pereira Moura Aranha, Vitor Augusto Dos Santos Garcia, Claudia Andrea Lima Cardoso, Silvia Maria Martelli

**Affiliations:** 1 Faculty of Exact Sciences and Technology, Federal University of Grande Dourados, Dourados 79804-970, MS, Brazil; igorgabrielsilvao@gmail.com (I.G.S.O.); morettonah@outlook.com (N.C.M.); mhelenaverdan@gmail.com (M.H.V.); 2 Faculty of Engineering, Federal University of Grande Dourados, Dourados 79804-970, MS, Brazil; karina-ueda@hotmail.com (K.S.U.F.); vininbds97@gmail.com (V.N.B.d.S.); carolinearanha@ufgd.edu.br (C.P.M.A.); 3 Faculty of Agricultural Sciences, São Paulo State University (UNESP), Av. Universitária, 3780, Botucatu 18610-034, SP, Brazil; vitor.as.garcia@unesp.br; 4 Center Studies in Natural Resources, State University of Mato Grosso do Sul, Postgraduate in Natural Resources, Dourados 79804-970, MS, Brazil; claudiacardosouems1@gmail.com

**Keywords:** annatto extract, edible coating, mulberry, shelf life

## Abstract

*Morus nigra* L., or mulberry, is a susceptible fleshy fruit due to its high respiratory rate and low storage stability, which shortens its shelf life and makes it difficult to commercialize in natura. Edible coatings, thin membranes produced directly on the desired surface, could improve food preservation, among other properties. Annatto (*Bixa orellana* L.) seeds are natural pigments with high antioxidant activity. This work aimed to develop a pectin-based edible coating with annatto extract to increase the shelf life of fruits, using mulberries as a study model. The mulberries were randomly separated into five groups: without coating, coated with different extract concentrations (0%, 5%, and 10%), and a layer-by-layer treatment consisting of a pectin layer under a 10% extract layer. The samples were evaluated for the following parameters: titratable acidity, maturity index, mass loss, pH, soluble solids, moisture contents, and bioactive compounds. The coated group with 10% annatto extract had the best result for the maturity index (25.52), while the group with 5% showed better mass loss and moisture (37.28% and 83.66%, respectively). Herein, it was demonstrated that pectin-based edible coatings with annatto extract delay the maturation and senescence of mulberries, preserving the bioactive compounds and increasing shelf life.

## 1. Introduction

*Morus nigra* L. (Moraceae), commonly known as black mulberry or silkworm mulberry, is an Asian species used in natura for human nutrition because of its high antioxidant activity due to its richness in phenolic compounds such as flavonoids and isoprenoids [1,2]. It is also considered an Unconventional Food Plant with potential applications in nutrition. The diversity of bioactive compounds present accounts for its antioxidant, anti-inflammatory, analgesic, and anti-tumor properties [3]. Mulberry is a fleshy fruit that is highly sensitive as a result of its elevated respiratory activity, causing a short shelf life and low stability during storage, thus making for difficult in natura marketing distribution [4]. Therefore, it is typically marketed in processed forms, such as jellies, pulps, and juices.

The biggest limitation in the entire mulberry production system is its rapid postharvest deterioration and limited shelf life, with losses reaching up to 70% [5]. For this reason, all the mulberry that needs to travel long distances should be made as frozen fruits or processed derivatives [6]. It is evident that alternatives are needed which would allow the fresh product to be marketed while preserving its quality properties.

Native to Brazil and other tropical regions of the globe, annatto (*Bixa orellana*, Bixaceae family) is well-known for its reddish seeds, rich in carotenoids (the main ones are bixin and norbixin) [7]. These seeds are used for food pigmentation in cheese, ice cream, margarine, and meat products [8]. Apart from a natural pigment, the compounds found in *B. orellana* (terpenes, steroids, carotenoids, saponins, tannins, alkaloids, flavonoids, quinones, polyphenols) present antioxidant and antimicrobial activities [9], offering a natural alternative for industries, whether in the form of extract or isolated compounds. These compounds extend the shelf life of products, delaying oxidative deterioration, as demonstrated by Stoll, Rech [10], who used bixin as an additive on active packaging to sunflower oil, presenting the best performance as an antioxidant agent compared to the other carotenoids tested.

Pectin is an anionic, amorphous, and colloidal carbohydrate component of plant cell walls, especially in mature fruits. Its complex structure constitutes the most intricate category of polysaccharides, primarily consisting of high-molecular-weight heterogeneous assemblies of glycanogalacturonans and acidic structural polysaccharides with varied compositions [11,12]. This is one of the most important polysaccharides, with remarkable cohesiveness, viscosity, and coating properties that have established it as a fundamental element in the conservation of various fruits and vegetables [13].

Due to its abundance, coating ability, biodegradability, and bioavailability, pectin represents a valuable natural resource commonly used in food packaging applications. It is classified according to its degree of methyl esterification as high methoxyl (HMP) or low methoxyl (LMP) pectin conformation; LMP is primarily used in coating technology [14]. Pectin-edible films have been enriched with *Morus alba* leaf extract, indicating that the incorporation of crude mulberry leaf extract and the bioactive components derived from them has greater biocompatibility and significantly improved mechanical and water barrier properties, which supports their suitability for food packaging/coating applications [15]. Pectin-edible films have also been incorporated with several antimicrobial substances such as nisin and essential oils, to obtain antimicrobial active packaging that contributes to extending product shelf life and reducing the risk of pathogen growth on food surfaces [16,17,18]. Pectin films have also been incorporated with *Ilex paraguariensis* (IP) extract. These films showed high amounts of bioactive compounds, a great potential to replace conventional primary packaging, and, if consumed with food as a bullet paper, they can add nutritional value to the packaged product [19]. The mechanical properties of pectin films enhanced with polyphenol-rich fruit extracts were investigated by Nastasi et al. The scavenging and reducing activity of plant extracts incorporated into the pectin films were determined using bench assays, and their antioxidant activity was correlated with a high presence of polyphenols, which predominantly comprised flavonoids and anthocyanins [20].

Edible coatings are thin membranes produced directly on the target surface. They possess properties that vary depending on the precursor solution. When applied to foods, these coatings act as active packaging, restraining and decreasing physical, chemical, and microbiological deterioration [21,22,23,24], maintaining quality, and extending the product’s shelf life. By incorporating additional components onto the coating (bioactive compound), such as essential oils, extracts, antimicrobials, and antioxidant agents, the physicochemical properties of the coating could be improved [21,25,26]. Thus, the protection of the fruit is raised, increasing the shelf life.

Accordingly, this study aimed to develop a pectin-based edible coating with annatto seed extract in order to extend the shelf life of small fruits, using mulberries (*Morus nigra*.) as the study model.

## 2. Materials and Methods

### 2.1. Materials

The chloroform (67-66-3) used was from the brand Qhemis (Jundiaí, Brazil), while D-Sorbitol P.S. (50-74-4) and citrus pectin (9000-69-5) were from Dinâmica^®^ (Indaiatuba, Brazil), acetone (67-64-1) from LSChemicals (Mumbai, India), ethanol (64-17-5) (Suzano, Brazil), sodium hydroxide (1310-73-2) from NEON (Suzano, Brazil), and Tween 20 (9005-64-5) from Proquimíos (Rio de Janeiro, Brazil).

### 2.2. Annatto Extract

Fresh seeds of *Bixa orellana* L. were obtained from the Lucinda Moretti Community Seed Bank (22°52′03.1″ S, 54°35′52.3″ W). Initially, annatto seeds were dried at 40 °C for 12 h. For the extract preparation, the seeds were continuously mixed at 1000 rpm and heated at 40 °C in chloroform for 60 min using a heating plate (IKA^®^ C-MAG HS 7, Staufen, Germany). The seeds were then separated from the solution through filtration, and subsequently, the solvent was removed under reduced pressure at 40 °C. Finally, the solvent residue was evaporated in a circulating oven (LUCA-82/221, Lucadema, São José do Rio Preto, Brazil) for 12 h at 40 °C.

### 2.3. Mulberries

Mulberries were harvested (22°12′11.1″ S 54°52′37.4″ W) and carefully selected to ensure uniform ripeness. Subsequently, they were cleaned using a 0.02% sodium hypochlorite solution, followed by drying. The samples were then assigned to one out of five treatments: uncovered (CT), covered with only a pectin coating (PEC), covered with annatto extract at 0.5% (PEC5), covered with annatto extract at 10% (PEC10), and a two-layer treatment in which a PEC10 layer was deposited above a PEC layer (PEC2L). The edible coatings were applied by spraying the solutions over the samples with the help of an atomizer.

For mass-loss analysis, mulberries were selected randomly and systematically labeled for consistent use across all experimental points. Each sample, weighing 50 g per treatment, was vacuum-sealed and stored in an ultra-low freezer (MDF-U5486SC Panasonic, Manaus, Brazil) at −54 °C. This storage condition was chosen to preserve sample integrity for the subsequent identification and quantification of active compounds.

### 2.4. Coating Solution

The precursor coating solution was prepared by dissolving 3% (*w*/*v* water) pectin in distilled water at 35 °C, supplemented with 20% (*w*/*w* of polymer) of D-sorbitol, which means for each 100 g of pectin, 20 g of sorbitol were used. The annatto seeds extract was incorporated into the solution along with the surfactant Tween 20 in a concentration of 5% (*w*/*w*) to maintain emulsion stability. The coating solution was agitated for 60 min to ensure proper mixing and homogeneity.

### 2.5. Titratable Acidity (TA), Soluble Solid Content (SSC), and Maturity Index (MI)

Titratable acidity was assessed by adding an aliquot (3 g) of the homogenized sample of fruits in 100 mL of distilled water, followed by titration with 0.1 N NaOH until reaching a pH endpoint of 8.2. The titratable acidity was expressed as grams of citric acid equivalent per 100 g of fruit, with determinations conducted in triplicate according to AOAC [16].

Soluble solids content (SSC) was determined following Method 315/IV of the Adolph Lutz Institute [17]. Samples were ground and homogenized, with analyses carried out in triplicate using a Carl Zeiss refractometer (Jena, Germany).

The maturity index (MI) was calculated as the ratio of soluble solids content to titratable acidity (MI = SSC/TA).

### 2.6. Mass Loss, Moisture Content, and Hydrogenionic Potential (pH)

The initial mass of the fruits was recorded at the onset of the experiment and, subsequently, daily throughout the storage period (12 days) using an analytical balance (Ohaus PA413P, Parsippany, NJ, USA). Mass loss was expressed as the percentage of initial total mass loss. Moisture content and total solids of the mulberries were determined in triplicate using an oven with forced ventilation (Lucadema, LUCA-82/221, São José do Rio Preto, Brazil) at 105 °C for 24 h. The pH determination was performed on triplicate at homogenized samples using a pHmeter (PH-2000, Instrutherm, São Paulo, Brazil), according to AOAC [18].

### 2.7. Bioactive Compounds

Carotenoid and anthocyanin analyses were conducted using a high-performance liquid chromatograph equipped with a quaternary pumping system (model LC-20AD, Shimadzu, Kyoto, Japan) coupled with a photodiode array detector (PDA) (model SPD-M20A, Shimadzu, Kyoto, Japan) and a time-of-flight mass spectrometer (QTof) (Bruker Daltonics, Billerica, MA, USA), with electrospray ionization and atmospheric pressure chemical ionization ion sources.

For anthocyanins, 1 g of mulberry was crushed and exhaustively extracted with acidified (HCl 1%) methanol in a homogenizer. The solutions were mixed and concentrated under reduced pressure, resulting in a crude anthocyanin extract. The anthocyanin identification was performed through HPLC-PDA-MS/MS, according to de Rosso and Mercadante (2007) [19]. The separation of anthocyanins was performed on a C18 Shim-pack CLC-ODS column (5 µm, 250 mm × 4.6 mm), eluted with a linear gradient of acidified water (5% formic acid) and methanol from 85:15 to 20:80 over 25 min, followed by a 15 min hold at this proportion, at a flow rate of 0.9 mL/min at 25 °C. Analyses were conducted at 520 nm, with spectra between 250 and 600 nm. Quantification was conducted using a cyanidin 3-glucoside standard curve in the concentration range from 5.00 to 20.00 µg/mL. Anthocyanins were identified based on UV-visible and mass spectra, compared with standards and literature data [20,21,22,23,24].

Carotenoid extraction from mulberries (1 g) was performed exhaustively with acetone, according to de Rosso and Mercadante (2007) [19]. Then, saponification was carried out with 10% KOH in methanol. The extract was concentrated under reduced pressure. Separation and identification of carotenoid through HPLC-PDA-MS/MS were made with a C30 YMC column (3 mm, 250 × 4.6 mm) using a liner gradient mobile phase of methanol/tert-buthyl methyl ether (TBME) from 95:5 to 70:30 in 30 min, then until 50:50 in 20 min, keeping this proportion for 35 min, with a flow rate of 0.9 mL/min, and a column temperature of 25 °C. The UV/vis spectra were recorded between 250 and 600 nm, and the analyses were performed at 450 nm. The carotenoids were identified considering the following information: elution order, coelution with standards, UV-vis and mass spectra characteristics compared to the literature [19,24,25,26]. A standard curve with all-trans-lutein and all-trans-carotenoid was prepared for quantification from 1.00 to 100.00 μg/mL. All samples were prepared in the mobile phase and filtered in PVDF (0.45 μm of pore size).

### 2.8. Statistical Analysis

Analysis of variance (ANOVA) was performed using Statistica^®®^ 5.5 software (Stasoft, Tulsa, OK, USA). Differences between treatments were determined using the Tukey test (*p* < 0.05).

## 3. Results

Pectin-based coatings are nontoxic, biodegradable, biocompatible with selective gas permeability, in addition to being transparent and resistant to oil and fats. They have high water vapor transmission rates due to hydrophilic nature kipping the sensory properties and quality of different fruits [27]. Below are presented the main results obtained from using edible pectin coatings in mulberry fruits.

### 3.1. Soluble Solid Content (SSC), Titratable Acidity (TA) and Maturity Index (MI)

During fruit maturation, it is normal for the total soluble solid content (SSC) to increase due to the degradation and biosynthesis of polysaccharides [28]. This phenomenon justifies the observed behavior of the SSC in all treatments, which gradually increased during the experiment, as presented in Table 1. The minor variations in initial Brix values among the treatments may be attributed to the addition of compounds from the coating solution, which are also soluble solids (e.g., pectin, sorbitol, and Tween 20) and may alter the composition of the fruit pulp. Consequently, this may affect the refractive index measured by the refractometer, leading to variations in the observed Brix values, in addition to variations inherent to the fruits themselves.

During the maturation process, as observed in all treatments, the concentration of simple sugars increased, while acidity decreased. Biosynthetic processes and polysaccharide degradation led to elevated SSC values, consequently enhancing the sweetness of the mulberries. This is due to the degradation of sucrose and reserve polysaccharides into fructose, which is responsible for the sweetness [29], and glucose, through the degradation of phosphorylated hexoses during respiration [30].

At the end of ripening, the highest sugar concentrations for the PEC, PEC5, and PEC10 treatments were on the 10th day, a trend not observed in the control (CT). After reaching the top, the SSC values tended to decrease, indicating the usage of sugars as an energy source [31]. This explains the decrease in the SSC values observed in the coated treatments during the final day of the experiment. A different pattern was observed for mulberries without coating, which kept increasing the SSC. From these results, it can be projected that, due to the high antioxidant capacity of annatto seed extract, it can protect the cellular structure of fruits and slow down oxidation processes [32]. Annatto seed is composed by a mixture of yellow-orange pigments resulting from the presence of several carotenoids, with an absolute predominance of an atypical one, known as bixin [33].

Together with this, the pectin coating also slows down the mass transport by reducing oxygen availability for metabolic processes. Jiménez-Villeda et al. have shown that pectin films demonstrated values for water vapor permeability (WVP) of 19.8 × 10^−11^ g m Pa^−1^ s^−1^ m^−2^ and oxygen permeability (OP) of 1.2 × 10^−12^ g m Pa^−1^ s^−1^ m ^−2^ [34]. Normally, pectin films containing plasticizers present high WVP numbers and low (OP), due to the hydrophilicity of pectin matrixes [35]. Amadeu et al. has shown that, among several variables studied, Sorbitol, the plasticizer, had the most significant influence on the permeability of the films [36]. Due to the soft texture of the fruit tissues of mulberries, they are easily prone to damage during storage and distribution, which can lead to a deterioration in quality and a loss of their commercial value. Considering that the coatings create a semi-permeable barrier to gases, this reduced oxygen availability, which slowed down the fruit’s metabolic processes. Pectin-edible coating can also help with possible mechanical damage.

During maturation, titratable acidity values are expected to decrease, as shown in Table 1, due to the oxidation of acids in the tricarboxylic acid cycle as a result of respiration. These acids are essential substrates in synthesizing volatile aromatics, phenolic compounds, and lipids [28,37,38]. Such chemical transformations during ripening and post-harvest are common in fruits, varying in intensity depending on the cultivar, storage methods, or maturation degree.

The maturation index (MI), calculated as the ratio of soluble solids content to titratable acidity, assesses the balance of sugars and acids within the pulp, a crucial aspect of fruit ripening. As expected, this index typically increases as fruits mature, reflecting the simultaneous increase in sugar content and decrease in acidity. This trend was observed across all treatments in the study. Notably, starting from the 10th day of experimentation, except for PEC5, the increase in the maturation index appeared to stabilize, and for the treatment with PEC2L a slight decline began, although not yet significant. This phenomenon suggests a potential shift from active maturation processes to metabolic pathways in energy production, marking the transition from ripening to post-ripening stages.

Upon completing the 12-day storage period, the CT sample exhibited the highest MI value. Significant differences were noted for the PEC5, PEC10, and PEC2L samples compared to the control, indicating that the extract reduced the fruits’ maturation index.

Flores et al., working with tomatoes, reported that pectin coatings with fiberelic acid were able to maintain firmness, delay weight loss, and decrease acidity, pH, sugar content, and color changes in fruits during 32 days of storage [21]. In another study, the authors demonstrated the feasibility of pectin coatings for extending the shelf life of fresh-cut pears [39], the results showing that pectin coating followed by dipping in solutions containing 2% CaCl_2_ was effective in controlling the weight loss and firmness of fresh-cut pears when compared to uncoated samples.

### 3.2. Mass Loss, Moisture Content, and pH

Weight loss is an indicator of freshness in fruits. Although various food commodities may experience mass loss differently, the primary cause of this phenomenon is typically moisture loss during fruit transpiration. Additionally, minor mass loss may occur due to carbon loss during gas exchange.

**Figure 1:**
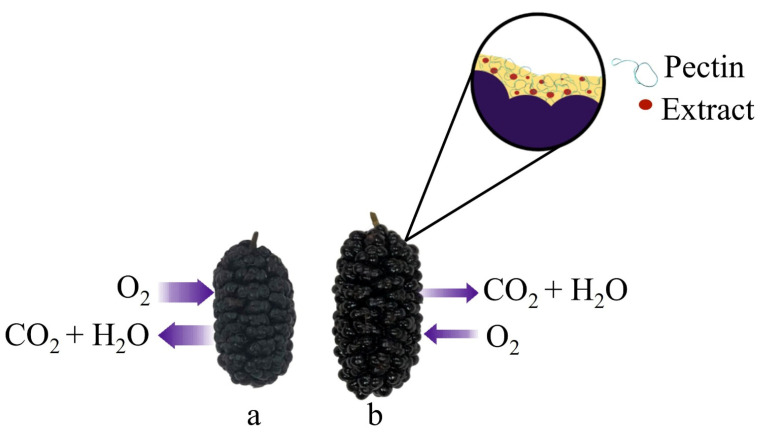
Schematic diagram of gas and water vapor permeability in the fruits of control treatment (**a**) and coating PEC5 (**b**).

**Figure 2:**
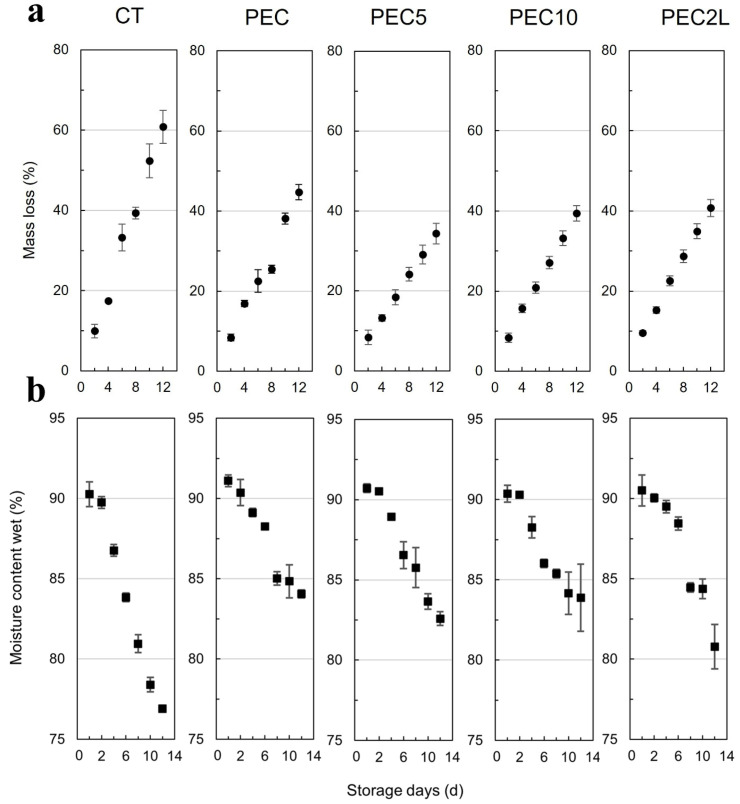
(**a**) Mass loss (%) during the 12 days of refrigerated storage, and (**b**) Moisture content of treatments during 12 days of refrigerated storage.

Edible coatings function as a secondary layer on fruit peel, acting as barriers to the entry and exit of compounds, thereby minimizing mass loss, as illustrated in Figure 1. The type of control infers how the hydrophilic/hydrophobic nature of coating compounds affects mass loss. Generally, losses occur more readily through hydrophilic bounds [40,41,42]. Upon analyzing average mass loss values, depicted in Figure 2a, it becomes apparent that all coating treatments exhibited lower percentages of mass loss than the control starting on day 4. By day 2 of the assay, the PEC5 treatment already displayed a significant difference compared to the CT. Ultimately, the percentage difference between coated treatments and the control ranged from 16.22% to 26.52% (PEC and PEC5, respectively).

The 5% extract (PEC5) treatment demonstrated the best performance, exhibiting statistical similarity to the final percentage of mass loss observed in the PEC10 and PEC2L treatments. Conversely, the control treatment presented the highest mass-loss rates, followed by the PEC sample. This suggests that the annatto extract, used as a bioactive compound in the pectin solution, enhances the barrier properties against mass loss during transpiration.

Despite the treatments with higher extract concentrations (PEC10 and PEC2L) exhibiting mass losses statistically similar to PEC5, there is a noticeable trend toward increasing percentages. This trend is evident considering that they are comparable to PEC, which experiences a mass loss 10% greater than PEC5. This behavior suggests that annatto extract, characterized by its hydrophobic nature, modifies the three-dimensional structure of pectin at higher concentrations, forming irregular regions (gaps) that promote moisture loss. This phenomenon is likely attributed to the decrease in adhesion between the two phases of the composite as the extract concentration in the solution increases, thereby negatively impacting the compatibility of the polymeric interface [43].

The results for mass loss of mulberries coated with pectin are consistent with the literature, as pectin can delay lipid migration and moisture loss, as well as improve the appearance of foods [44,45].

Figure 2b presents the samples’ moisture during storage, reinforcing the mass loss results and demonstrating the coatings’ ability as an external barrier to minimize both moisture and mass losses of the fruits. This is evident as the CT already had moisture below 85% on the 6th day of storage, ending the experiment with an average of 78.40%. In contrast, the other treatments maintained averages between 84.84% and 83.66% on the last day.

Pectin is a polymer able to produce hydrogels [46] capable of absorbing and retaining water, forming a highly hydrophilic matrix surrounding the fruits. Pectin-based gel is a three-dimensional network structure consisting of a gel backbone (formed by pectin alone or combined with other biopolymers) and liquid phase (water, emulsion, etc.), which is a biopolymer soft colloidal material [47]. This explains the reduction in mass loss of mulberries coated with pectin in relation to the control sample.

The pH values, as well as the soluble solids content, increased during the evaluated period (Table 2) due to chemical transformations that occur with ripening, such as acid oxidation and synthesis of volatile and phenolic compounds [28,37,48].

In the final days of analysis, a decline in pH is observed, likely attributed to metabolite degradation, affecting the values of SSC and titratable acidity. The pH values ranged from 3.15 to 4.19, consistent with previous findings by Ercisli and Orhan [49], who reported pH values of 5.60, 3.52, and 4.04 for three species of the genus *Morus*, *M. alba*, *M. nigra*, and *M. rubra*, respectively. Notably, significant differences were observed only for *M. alba*. Mota [50] similarly observed pH values ranging from 3.26 to 3.47 in blackberries from seven *Rubus* sp. cultivars, aligning closely with our results. These pH variations, compared to the values in the literature, may reflect the plant’s inherent characteristics, the blackberries’ harvesting season, or even the type and quality of the soil.

Towards the end of the experiment, specific treatments, such as CT and PEC5, maintained pH values statistically similar to those at the beginning despite fluctuations throughout the storage period. This phenomenon may be attributed to the buffering capacity exhibited by some juices, resulting in apparent chemical variations in acidity but minimal changes in pH values [28].

### 3.3. Bioactive Compounds

The anthocyanin content is presented in Table 3. Cyanidin 3-glucoside was quantified as the major anthocyanin, comprising 91.1% of the total anthocyanins, consistent with the anthocyanin profiles reported by other authors such as Wu and Prior [51], who obtained values ranging from 88.6% to 95.2%, and Ferreira, Rosso [52], who reported a value of 92.9%.

In the determination of carotenoids, the major compounds were all-*trans*-carotene (38.7% on day zero) and all-*trans*-lutein (29.4% on day zero) concerning total carotenoids, as shown in Table 3, which is also consistent with findings reported by Ferreira, Rosso [52].

The degradation profile of total bioactive compounds in coated or uncoated mulberry samples over the 12-day storage period at 5 °C is shown in Table 3 and Table 4. Notably, edible coatings protect the active compounds in all coated samples, resulting in lower degradation than the control mulberries without coating. Notably, adding annatto extract, rich in pigments and antioxidant compounds, to the pectin films contributed to preserving the bioactive compounds in the mulberries for an extended duration.

By the end of the 12-day storage period, the control mulberries had experienced a 23% loss of all their active compounds. In contrast, the pectin and annatto extract-coated samples exhibited less than 20% losses (Table 5). This indicates superior performance in preserving the active compounds, particularly with higher concentrations of annatto extract (PEC5 maintaining 84% and PEC10 87%). The treatment involving two layers of coatings (PEC2L) showed a 10% loss of the active compounds.

## 4. Conclusions

Despite the complexity of studying mulberries due to their high sensitivity and rapid post-harvest metabolism, our findings demonstrate that the application of edible coatings composed of pectin and annatto extract effectively delays the ripening and senescence of *M. nigra* mulberries, thereby extending their shelf life. Remarkably, the inclusion of annatto extract, especially at a concentration of 5% (*w*/*w* pectin), enhanced the capacity of the pectin-based coating to act as a selective barrier, minimizing mass losses and slowing down the overall metabolism of mulberries.

## Figures and Tables

**Table 1:** Mean values of soluble solids content (SSC) (%), titratable acidity (g/kg of citric acid), and maturation index during 12 days of storage.

SSC (Brix %)					
Day	CT	PEC	PEC5	PEC10	PEC2L
0	7.00 ^Ag^ ± 0.01	7.00 ^Ae^ ± 0.01	7.50 ^Af^ ± 0.01	7.00 ^Ag^ ± 0.01	7.50 ^Ag^ ± 0.01
2	8.00 ^Af^ ± 0.01	7.00 ^Ce^ ± 0.01	7.00 ^Cf^ ± 0.01	7.50 ^Bf^ ± 0.01	8.00 ^Af^ ± 0.01
4	11.00 ^Ae^ ± 0.01	9.50 ^Cd^ ± 0.01	8.00 ^Ee^ ± 0.01	10.50 ^Be^ ± 0.01	9.00 ^De^ ± 0.01
6	17.50 ^Ad^ ± 0.01	9.50 ^Cd^ ± 0.01	9.50 ^Cd^ ± 0.01	9.50 ^Cd^ ± 0.01	12.50 ^Bd^ ± 0.01
8	18.00 ^Ac^ ± 0.01	12.50 ^Cc^ ± 0.01	11.75 ^Dc^ ± 0.01	11.50 ^Dc^ ± 0.01	13.25 ^Bc^ ± 0.01
10	19.50 ^Ab^ ± 0.01	17.00 ^Bb^ ± 0.01	16.00 ^Ca^ ± 0.01	15.00 ^Db^ ± 0.01	15.00 ^Db^ ± 0.01
12	21.50 ^Aa^ ± 0.01	13.50 ^Da^ ± 0.01	14.50 ^Cb^ ± 0.01	14.00 ^Ca^ ± 0.01	17.50 ^Ba^ ± 0.01
Titratable Acidity (g/kg of Citric Acid)					
Day	CT	PEC	PEC5	PEC10	PEC2L
0	11.72 ^Aa^ ± 0.07	11.53 ^Aa^ ± 0.06	11.09 ^Ab^ ± 0.04	10.17 ^Ab^ ± 0.05	12.02 ^Aa^ ± 0.09
2	8.58 ^Bb^ ± 0.06	8.40 ^Bb^ ± 0.05	13.27 ^Aa^ ± 0.03	13.73 ^Aa^ ± 0.01	9.00 ^Bb^ ± 0.01
4	6.27 ^Bc^ ± 0.04	8.24 ^Ab^ ± 0.03	8.43 ^Ac^ ± 0.03	7.12 ^ABcd^ ± 0.12	6.70 ^Bc^ ± 0.05
6	5.77 ^Bc^ ± 0.01	7.94 ^Ab^ ± 0.07	7.07 ^ABd^ ± 0.10	5.82 ^Bd^ ± 0.11	6.09 ^Bcde^ ± 0.05
10	6.04 ^Bc^ ± 0.06	4.33 ^Cc^ ± 0.05	7.74 ^Acd^ ± 0.03	6.19 ^Bd^ ± 0.05	5.33 ^BCe^ ± 0.09
12	5.62 ^Bc^ ± 0.04	4.95^Cc^ ± 0.04	5.57 ^Ce^ ± 0.02	6.66 ^Acd^ ± 0.01	5.60 ^Bde^ ± 0.04
Maturation Index					
Day	CT	PEC	PEC5	PEC10	PEC2L
0	6.05 ^Af^ ± 0.36	6.06 ^Ae^ ± 0.19	6.76 ^Ae^ ± 0.11	6.40 ^Ad^ ± 0.58	6.22 ^Ae^ ± 0.16
2	9.35 ^Ae^ ± 0.66	8.35 ^Bd^ ± 0.47	5.28 ^Cf^ ± 0.13	5.46 ^Cd^ ± 0.053	8.89 ^Abd^ ± 0.09
4	17.58 ^Ad^ ± 1.03	11.54 ^Cc^ ± 0.40	9.50 ^Dd^ ± 0.33	13.55 ^Bc^ ± 1.22	13.45 ^BCc^ ± 0.95
6	30.32 ^Ab^ ± 0.61	12.01 ^Dc^ ± 0.98	14.89 ^Cc^ ± 0.41	18.99 ^Bb^± 0.61	19.63 ^Bb^ ± 0.81
10	31.59 ^Ab^ ± 0.80	31.12 ^Ab^ ± 0.47	15.19 ^Cc^ ± 0.55	17.89 ^Cb^ ± 0.73	27.36 ^Ba^ ± 2.19
12	33.43 ^Aa^ ± 0.18	32.92 ^Aab^ ±1.36	28.72 ^Ba^± 0.89	22.52 ^Da^ ± 0.30	25.88 ^Ca^± 1.57

CT—control sample, PEC—coating with only pectin, PEC5—coating with 5% of annatto extract in pectin, PEC10—coating with 10% of annatto extract in pectin, PEC2L—coating with one layer of pectin coating under a layer of PEC10. Means followed by the same letter do not differ from each other at a 5% probability level (*p* > 0.05) by Tukey’s test. Capital letters compare means horizontally (between treatments on the same day), while lowercase letters compare means vertically (between days in the same treatment).

**Table 2:** Mean pH values per treatment during the storage period.

pH					
Day	CT	PEC	PEC5	PEC10	PEC2L
0	3.30 ^Ab^ ± 0.05	3.30 ^Ad^ ± 0.05	3.30 ^Aef^ ± 0.05	3.30 ^Ad^ ± 0.05	3.30 ^Ae^ ± 0.05
2	3.51 ^Ab^ ± 0.03	3.37 ^Bd^ ± 0.03	3.15 ^Df^ ± 0.01	3.23 ^Cd^ ± 0.01	3.32 ^Be^ ± 0.04
4	4.07 ^Aa^ ± 0.03	3.84 ^Bb^ ± 0.03	3.59 ^Ccd^ ± 0.01	4.04 ^Aa^ ± 0.07	3.92 ^Ba^ ± 0.03
6	4.14 ^Aa^ ± 0.26	3.70 ^Bb^ ± 0.11	3.73 ^ABbc^ ± 0.20	3.77 ^ABb^ ± 0.18	3.53 ^Bd^ ± 0.04
8	4.19 ^Aa^ ± 0.17	4.12 ^Aa^ ± 0.10	3.78 ^Bab^ ± 0.02	3.87 ^Bb^ ± 0.03	3.82 ^Bb^ ± 0.06
10	4.11 ^Aa^ ± 0.07	3.79 ^Cb^ ± 0.03	3.95 ^Ba^ ± 0.03	3.54 ^Ec^ ± 0.02	3.68 ^Dc^ ± 0.01
12	3.55 ^BCb^ ± 0.04	3.75 ^Abc^ ± 0.05	3.43 ^Cde^ ± 0.08	3.60 ^Bc^ ± 0.06	3.51 ^BCd^ ± 0.01

Means followed by the same letter do not differ from each other at a 5% probability level (*p* > 0.05) by Tukey’s test. Capital letters compare means horizontally (between treatments on the same day), while lowercase letters compare means vertically (between days in the same treatment).

**Table 3:** Amount of cyanidin 3-glucoside (%) in relation to total anthocyanins in mulberries during 12 days of storage at 6 °C.

Treatment		Storage Days			
	0	3	6	9	12
CT	91.1 ^Aa^ ± 0.2	82.9 ^Bd^ ± 0.1	81.1 ^Cd^ ± 0.1	76.7 ^De^ ± 0.2	70.2 ^Ee^ ± 0.1
PEC	91.1 ^Aa^ ± 0.2	84.7 ^Bc^ ± 0.1	83.0 ^Cc^ ± 0.0	79.3 ^Dd^ ± 0.2	74.7 ^Ed^ ± 0.2
PEC5	91.1 ^Aa^ ± 0.2	85.0 ^Bc^ ± 0.1	83.1 ^Cc^ ± 0.2	80.2 ^Dc^ ± 0.1	76.6^Ec^ ± 0.1
PEC10	91.1 ^Aa^ ± 0.2	85.6 ^Bb^ ± 0.2	83.8 ^Cb^ ± 0.1	81.1 ^Db^ ± 0.1	79.3 ^Eb^ ± 0.2
PEC2L	91.1 ^Aa^ ± 0.2	86.6 ^Ba^ ± 0.2	85.6 ^Ca^ ± 0.2	82.9 ^Da^ ± 0.2	82.0 ^Da^ ± 0.1

Means followed by the same letter do not differ from each other at a 5% probability level (*p* > 0.05) by Tukey’s test. Capital letters compare means horizontally (between treatments on the same day), while lowercase letters compare means vertically (between days in the same treatment).

**Table 4:** Content of all-*trans*-carotene and all-*trans*-lutein (%) in relation to the total carotenoids in mulberries during 12 days of storage at 6 °C.

all-*trans*-carotene					
Treatment	Storage days				
	0	3	6	9	12
CT	38.7 ^Aa^ ± 0.2	35.4 ^Bd^ ± 0.0	34.5 ^Cd^ ± 0.0	32.6 ^De^ ± 0.1	29.17 ^Ec^ ± 0.55
PEC	38.7 ^Aa^ ± 0.2	36.1 ^Bc^ ± 0.1	35.3 ^Cc^ ± 0.2	33.7 ^Dd^ ± 0.1	31.7 ^Eb^ ± 0.2
PEC5	38.7 ^Aa^ ± 0.2	36.3 ^Bbc^ ± 0.2	35.4 ^Cbc^ ± 0.1	34.1 ^Dc^ ± 0.2	32.6 ^Eb^ ± 0.2
PEC10	38.7 ^Aa^ ± 0.2	36.4 ^Bb^ ± 0.1	35.6 ^Cb^ ± 0.0	34.5 ^Db^ ± 0.2	33.7 ^Ea^ ± 0.3
PEC2L	38.7 ^Aa^ ± 0.2	36.7 ^Ba^ ± 0.2	36.5 ^Ba^ ± 0.2	35.4 ^Ca^ ± 0.1	34.9 ^Da^ ± 0.2
all-*trans*-lutein					
Treatment	Storage days				
	0	3	6	9	12
CT	29.4 ^Aa^ ± 0.3	26.9 ^Bc^ ± 0.2	26.2 ^Cd^ ± 0.1	24.7 ^Dc^ ± 0.1	22.6 ^Ee^ ± 0.2
PEC	29.4 ^Aa^ ± 0.3	27.3 ^Bbc^ ± 0.2	26.7 ^Cc^ ± 0.1	25.7 ^Db^ ± 0.1	24.1 ^Ed^ ± 0.3
PEC5	29.4 ^Aa^ ± 0.3	27.4 ^Bb^ ± 0.1	26.9 ^Cbc^ ± 0.25	25.9 ^Db^ ± 0.2	24.7 ^Ec^ ± 0.1
PEC10	29.4 ^Aa^ ± 0.3	27.7 ^Bab^ ± 0.3	27.0 ^Cb^ ± 0.1	26.2 ^Db^ ± 0.3	25.6 ^Eb^ ± 0.0
PEC2L	29.4 ^Aa^ ± 0.3	27.9 ^Ba^ ± 0.1	27.6 ^BCa^ ± 0.3	26.6 ^Ca^ ± 0.1	26.5 ^Ca^ ± 0.2

Means followed by the same letter do not differ from each other at a 5% probability level (*p* > 0.05) by Tukey’s test. Capital letters compare means horizontally (between treatments on the same day), while lowercase letters compare means vertically (between days in the same treatment).

**Table 5:** Decrease in the total concentration of active compounds in mulberries during 12 days of storage at 6 °C.

Treatments	Storage Days				
	0	3	6	9	12
CT	100.0	91.0	89.0	84.0	77.0
PEC	100.0	93.0	91.0	87.0	82.0
PEC5	100.0	93.0	91.0	88.0	84.0
PEC10	100.0	94.0	92.0	89.0	87.0
PEC2L	100.0	95.0	94.0	91.0	90.0

## Data Availability

No data were used for the research described in the article.

## Abbreviations

The following abbreviations are used in this manuscript:

CT	Control sample
MI	Maturity Index
PEC	Coating with only pectin
PEC5	Coating with 5% of annatto extract in pectin
PEC10	Coating with 10% of annatto extract in pectin
PEC2L	Coating with one layer of pectin coating under a layer of PEC10
SSC	Soluble solids content
TA	Titratable acidity
